# Forehead Reduction Using the Subsuperficial Galea Dissection Plane

**DOI:** 10.7759/cureus.115470

**Published:** 2026-08-30

**Authors:** Malaz S Younis, Ali S Hamid, Mostafa Gamal Khalaf, Asjad Bahaeldin Idris, Mazin Abdelrahman Elhag, Faleh Al-Shahrani

**Affiliations:** 1 Surgery, King Saud Medical City, Riyadh, SAU; 2 Plastic Surgery, Dr. Mohammed Alfaqih Hospital, Riyadh, SAU; 3 Cosmetic Surgery, Dr. Mohammed Alfaqih Hospital, Riyadh, SAU; 4 Surgery, King Fahad Medical City, Riyadh, SAU

**Keywords:** contour irregularity, flap thickness, forehead reduction, frontalis muscle, hairline lowering, scalp advancement, subgaleal dissection, supra frontalis plane

## Abstract

Forehead reduction with hairline advancement is a well-established procedure for correcting a congenitally high or surgically elevated hairline. The standard surgical approach, described consistently throughout the published literature, involves elevation of the scalp flap in the subcutaneous plane. The frontalis muscle varies considerably in width, depth, and presence across the forehead and fades gradually at the central bifurcation found in a substantial proportion of individuals. In some cases, this may cause the inadvertent elevation of parts of the muscle when performing multiple deep tissue manipulations involving the frontalis muscle. We present a technique for reliably maintaining a single, consistent dissection plane (subsuperficial galea) throughout the forehead, to produce a flap of uniform thickness and composition that we hypothesize may reduce postoperative contour irregularity after multiple deep plane manipulations. Following standard incision and hairline marking, dissection is carried in the subsuperficial galea plane immediately superficial to the frontalis muscle, using the muscle's characteristic glistening, striated surface as a continuous landmark. Through the midline bifurcation, where this landmark is absent, plane depth is maintained by reference to the plane already established on either side and by tactile assessment of tissue thickness and tension. The supraorbital and supratrochlear neurovascular bundles are identified as they perforate the frontalis muscle and are preserved as dissection approaches the brow. Deliberately and consistently maintaining the dissection plane (subsuperficial galea plane) throughout the forehead, with specific attention to the bifurcation zone, produces a flap of uniform thickness and, based on our clinical impression, may reduce the visibility of postoperative contour irregularities. We present the anatomical rationale and step-by-step technique to allow evaluation and reproduction by other surgeons.

## Introduction

Forehead reduction with hairline advancement is an established aesthetic procedure for patients with a congenitally high hairline, a hairline that has receded due to early androgenic alopecia, or postsurgical hairline deformity following previous hair transplantation [1,2]. The procedure involves elevation of the forehead and anterior scalp flap, advancement of the scalp forward, and excision of the resulting excess forehead skin to bring the hairline to a more aesthetically favourable position [1]. Despite the procedure's increasing popularity, including its frequent use in facial feminisation surgery, descriptions of the surgical technique throughout the published literature are remarkably consistent in one respect: virtually all reported series describe scalp/forehead flap elevation in the subcutaneous plane.

Reported modifications of this standard approach have generally focused on incision design, hairline irregularity, and adjunctive procedures, such as scalp reduction or brow lifting, rather than on the composition or thickness of the elevated flap itself. Postoperative contour irregularity following forehead reduction has not, to our knowledge, been specifically reported or systematically studied as a distinct complication in the existing literature. We reason that a flap of uniform composition and thickness should redrape more predictably over the recipient bed than one with regional variation, and it is this specific link - between dissection-plane consistency and contour irregularity - that has not previously been examined in forehead reduction, and that the present technique aims to address.

This plane is favoured because it is relatively avascular, technically straightforward to develop, and provides excellent scalp mobility for posterior dissection and advancement [1]. However, achieving this plane consistently across the entire forehead is more technically demanding than its description in the literature suggests. The frontalis muscle does not have a uniform distribution: anatomical studies have demonstrated that it is broadest and thickest centrally, frequently interrupted by a central bifurcation in a substantial proportion of individuals, and thins progressively toward the temporal regions [3]. Because the muscle's glistening, striated surface is the principal landmark by which the correct dissection plane is recognised, regions where the muscle is thin, absent at the bifurcation, or transitioning into adjacent muscles provide a less reliable landmark, and the dissection plane can drift deeper than intended without this being immediately apparent to the surgeon.

In our clinical experience, this inconsistency becomes most apparent after flap advancement and redraping. Variable-depth dissection produces a flap with regional differences in soft-tissue composition and thickness. During closure, these differences may create localized variations in the way the flap conforms to the underlying recipient bed. Particularly in patients with thin soft tissues, these transitions may become visible as subtle contour irregularities despite otherwise successful hairline advancement. We therefore believe that maintaining a uniformly thick flap is itself an important technical objective, independent of whether the frontalis muscle is preserved.

We describe a technique for maintaining a single, consistent dissection plane superficial to the frontalis muscle (subsuperficial galea plane) across the entire forehead, including through the region, principally the central bifurcation, where the muscle itself cannot be used as a landmark. By maintaining this consistency, the flap comprises the same tissue layers (skin, subcutaneous fat, and subsuperficial galea) throughout its extent, while preserving the frontalis muscle completely undisturbed on the recipient bed. We present the anatomical basis for this approach and a detailed, reproducible description of the surgical technique, informed by our clinical experience using it in forehead reduction surgery at our centre.

## Technical report

Surgical technique

Patient Selection and Preoperative Markings

Patients suitable for this technique are those with an indication for standard hairline-lowering surgery: a congenitally high hairline, hairline recession amenable to surgical correction, or post-surgical deformity from previous hair restoration procedures [1,2]. Preoperative markings follow standard hairline-lowering principles: the existing hairline is marked in its natural, irregular pattern, and the planned new hairline position is marked anteriorly, with the distance of advancement determined by preoperative assessment of scalp laxity [1].

Incision

An incision is made along the marked anterior hairline, bevelled to follow the natural angle of hair follicle emergence to optimise postoperative hair growth through the scar, consistent with standard hairline-lowering technique [1]. The incision is carried through skin layers and subcutaneous tissue (Figure 1).

**Figure 1:**
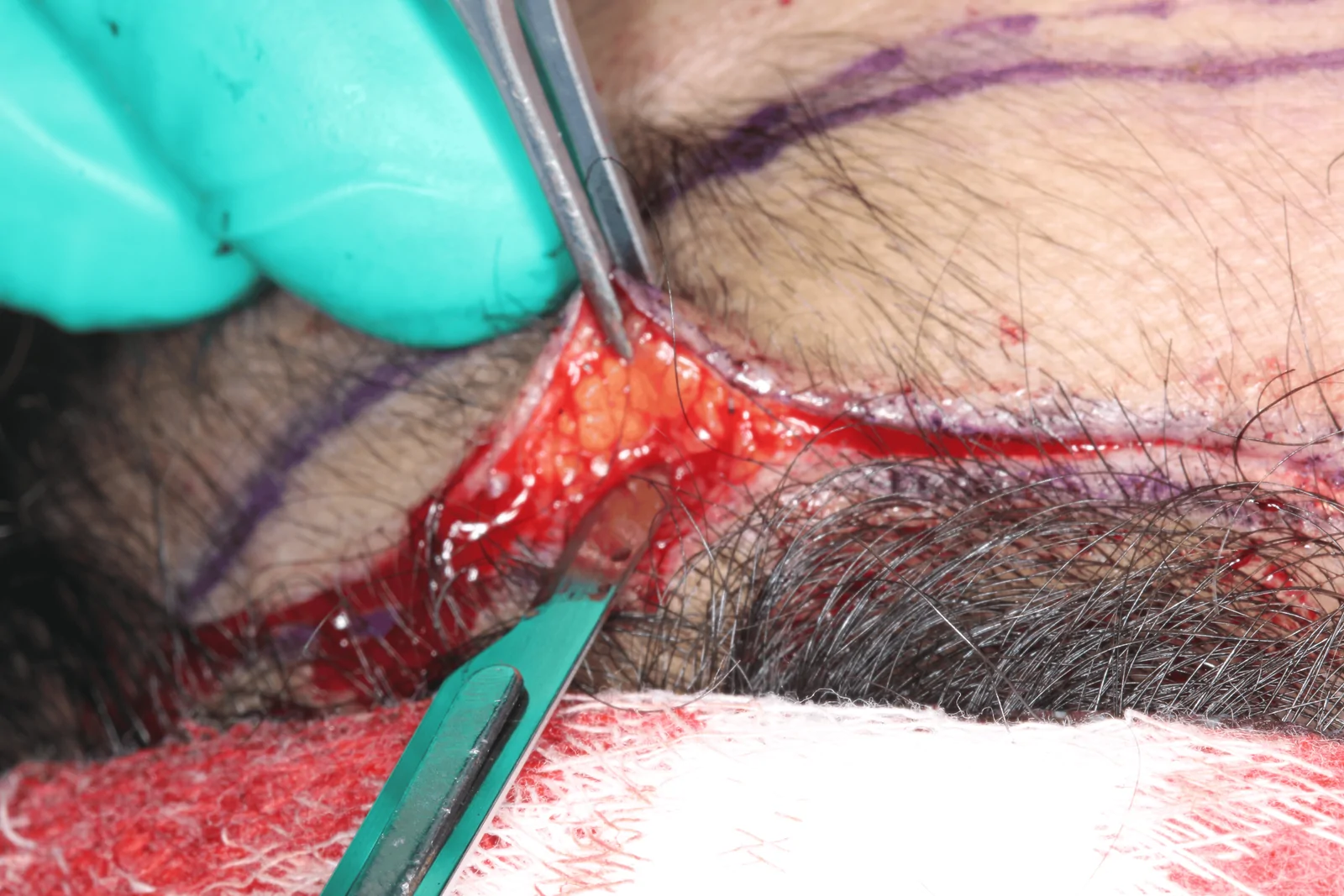
Anatomical Dissection Along the Hairline Incision Detailed intraoperative view of the hairline incision. Careful visualization at this stage facilitates safe tissue elevation while minimizing the risk of inadvertent injury.

*Maintaining the Subsuperficial Galea Plane* 

Following incision, dissection is carried out in the subsuperficial galea plane immediately superficial to the frontalis muscle, identified by its characteristic glistening and striated appearance, and is deliberately maintained at a consistent depth across the forehead rather than allowed to drift deeper in regions where this landmark is less distinct. Throughout the dissection, attention is directed not only toward remaining superficial to the frontalis muscle but also toward maintaining a constant flap thickness. The objective is to elevate a flap with identical tissue composition from edge to edge rather than one containing alternating regions of differing thickness created by inadvertent variation in dissection depth (Figure 2). This prevents postoperative contour irregularities that may appear after deep tissue manipulations, such as prominent frontal bone shaving, where the frontalis muscle is divided in a longitudinal manner up to the periosteum, providing direct access for bone shaving. Dissection proceeds using a combination of sharp dissection with facelift scissors at the outset, transitioning to trepsat forceps, and then blunt finger dissection once the correct fascial plane is entered [4]. 

**Figure 2:**
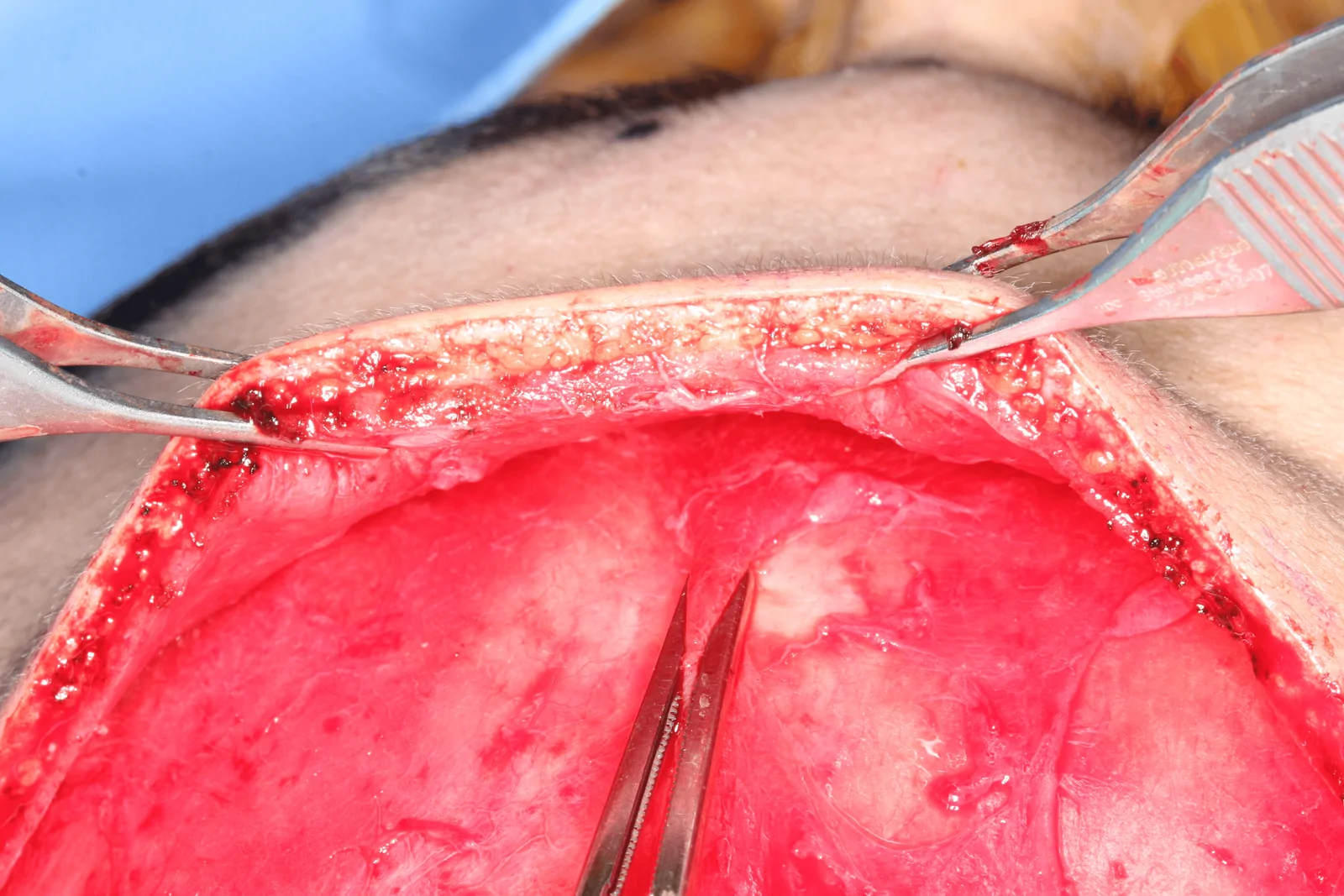
Forehead Tissue Layers and Flap Thickness Intraoperative exposure of the forehead following flap elevation, demonstrating the layered anatomy of the flap, showing the skin, subcutaneous fat, and the superficial galea. The frontalis muscle and the underlying deep galea are intact and help with the deBakey forceps.

Particular care is required in the midline, where the frontalis muscle bifurcates or is deficient in a substantial proportion of patients, removing the muscle landmark relied upon elsewhere to confirm dissection depth. In this region, plane depth is maintained by continuity with the plane already established laterally, where the muscle is present and visible, and by tactile assessment of tissue thickness and resistance rather than by direct visualisation of muscle. Working from lateral to medial, so that the correct plane is established on both sides before the bifurcation zone is reached, helps avoid inadvertent deepening in this area. The dissection is carried anteriorly to the extent required for adequate skin mobilisation and advancement, remaining superficial to the frontalis muscle throughout the forehead.

Frontal Bone Contouring 

Following elevation of the scalp flap in the subgaleal plane and based on the preoperative markings and intraoperative assessment, the corresponding region of the frontalis muscle was identified (Figure 3). A longitudinal incision was made through the frontalis muscle directly over the planned contouring site, and the muscle fibres were gently elevated to expose the underlying frontal bone while preserving the surrounding soft tissues.

**Figure 3:**
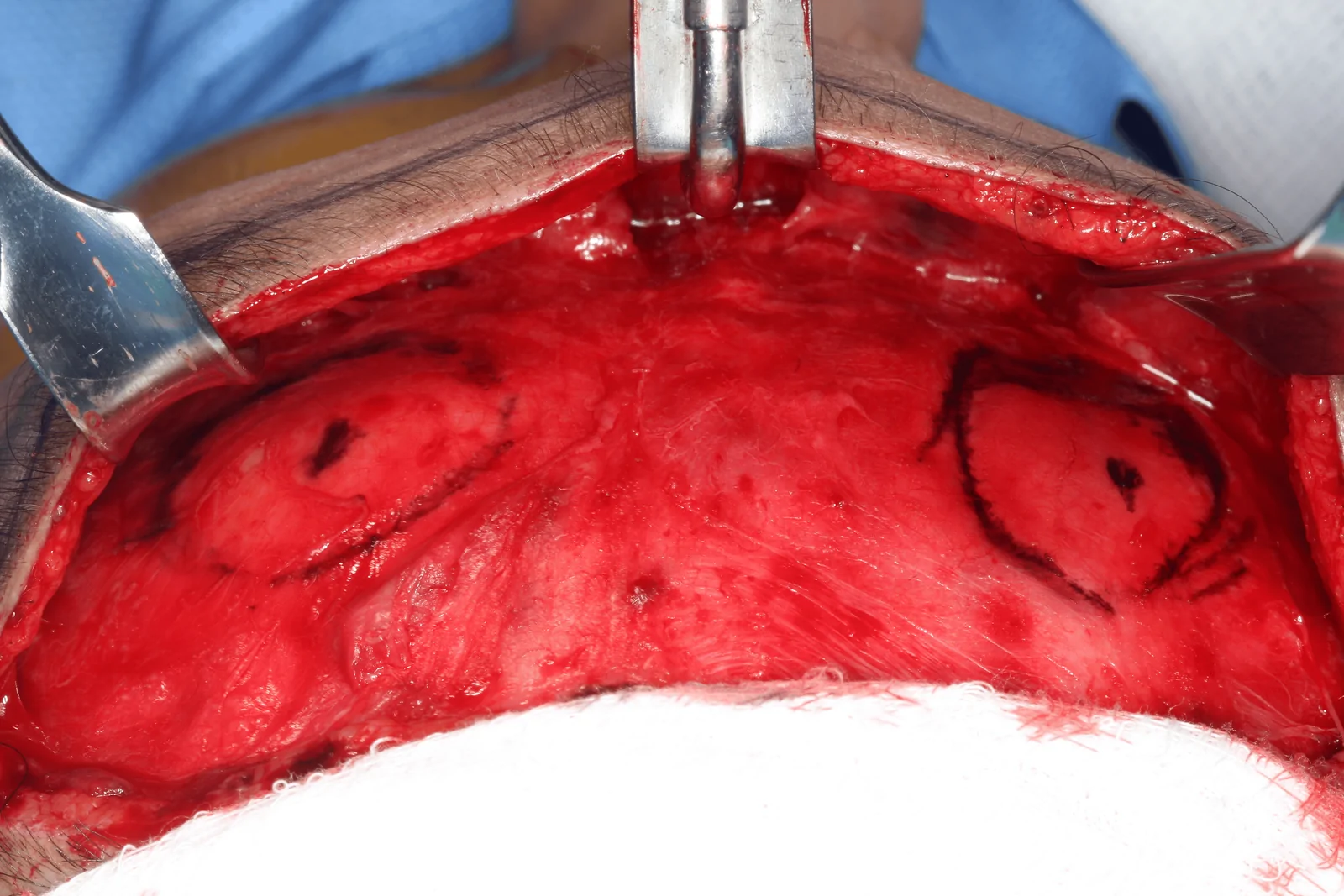
Surface Markings on the Frontalis Muscle Bed for Frontal Bone Reduction Intraoperative view of the frontalis muscle bed, demonstrating the surface markings used to identify the planned areas of frontal bone contouring prior to muscle incision and bone shaving.

Using a high-speed surgical burr with continuous saline irrigation, the prominent cortical bone was carefully reduced in a controlled and progressive manner until the desired contour was achieved (Figure 4). Bone removal was performed conservatively, with frequent reassessment of the forehead profile to maintain symmetry and avoid excessive reduction. Particular attention was paid to preserving the integrity of the outer cortical table and avoiding unnecessary thinning of the frontal bone.

**Figure 4:**
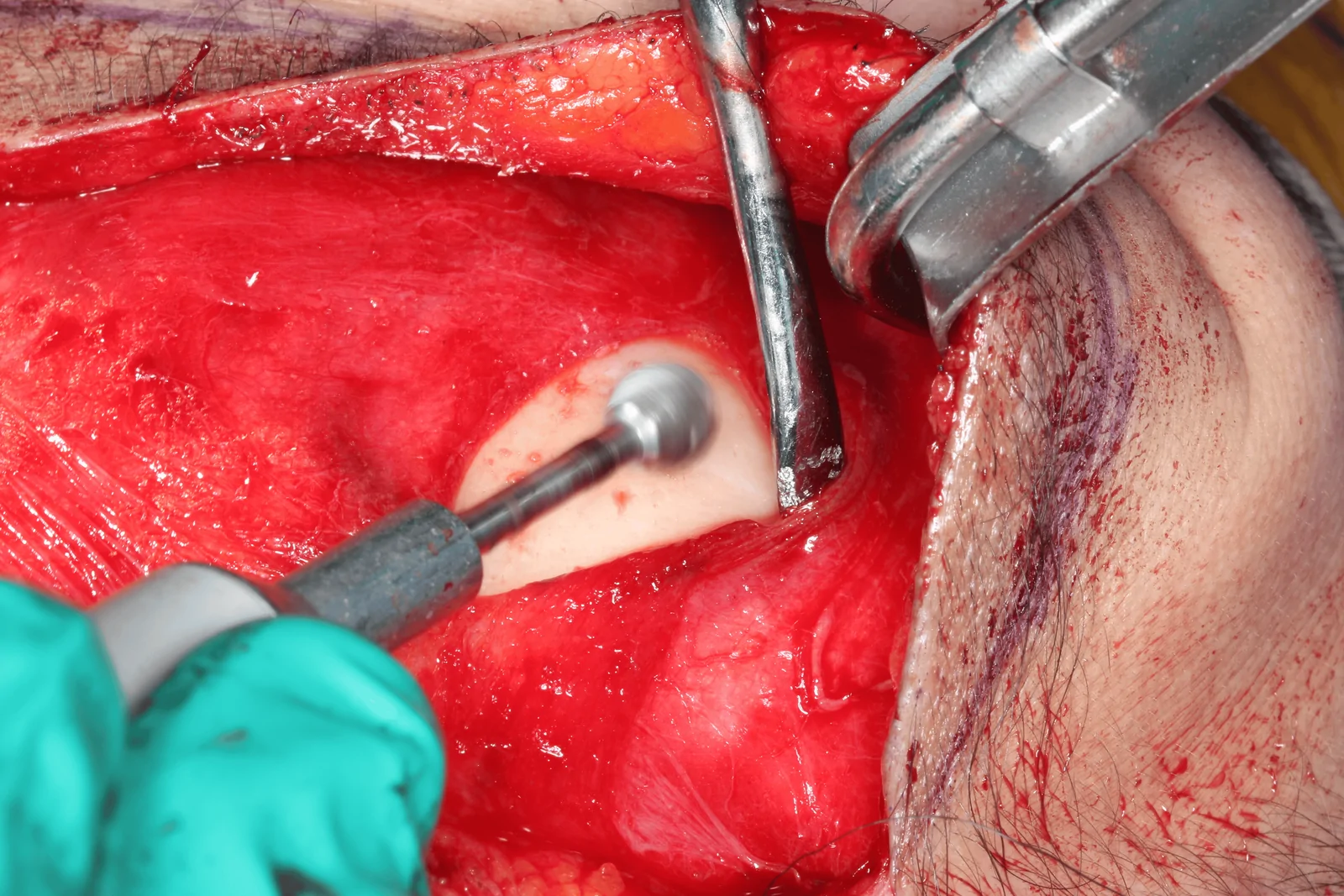
Frontal Bone Contouring Following Frontalis Muscle Elevation Intraoperative view demonstrating frontal bone contouring after incision and elevation of the frontalis muscle. A high-speed surgical burr was used to reduce the prominent frontal cortical bone under continuous irrigation, allowing controlled contour refinement while preserving the surrounding soft tissues.

Following completion of the osseous contouring, the operative field was irrigated thoroughly to remove bone debris and achieve meticulous haemostasis. The divided frontalis muscle was then re-approximated anatomically using sutures, restoring continuity of the muscular layer and completely covering the contoured bone (Figure 5). The procedure then proceeded with the planned forehead reduction and layered wound closure.

**Figure 5:**
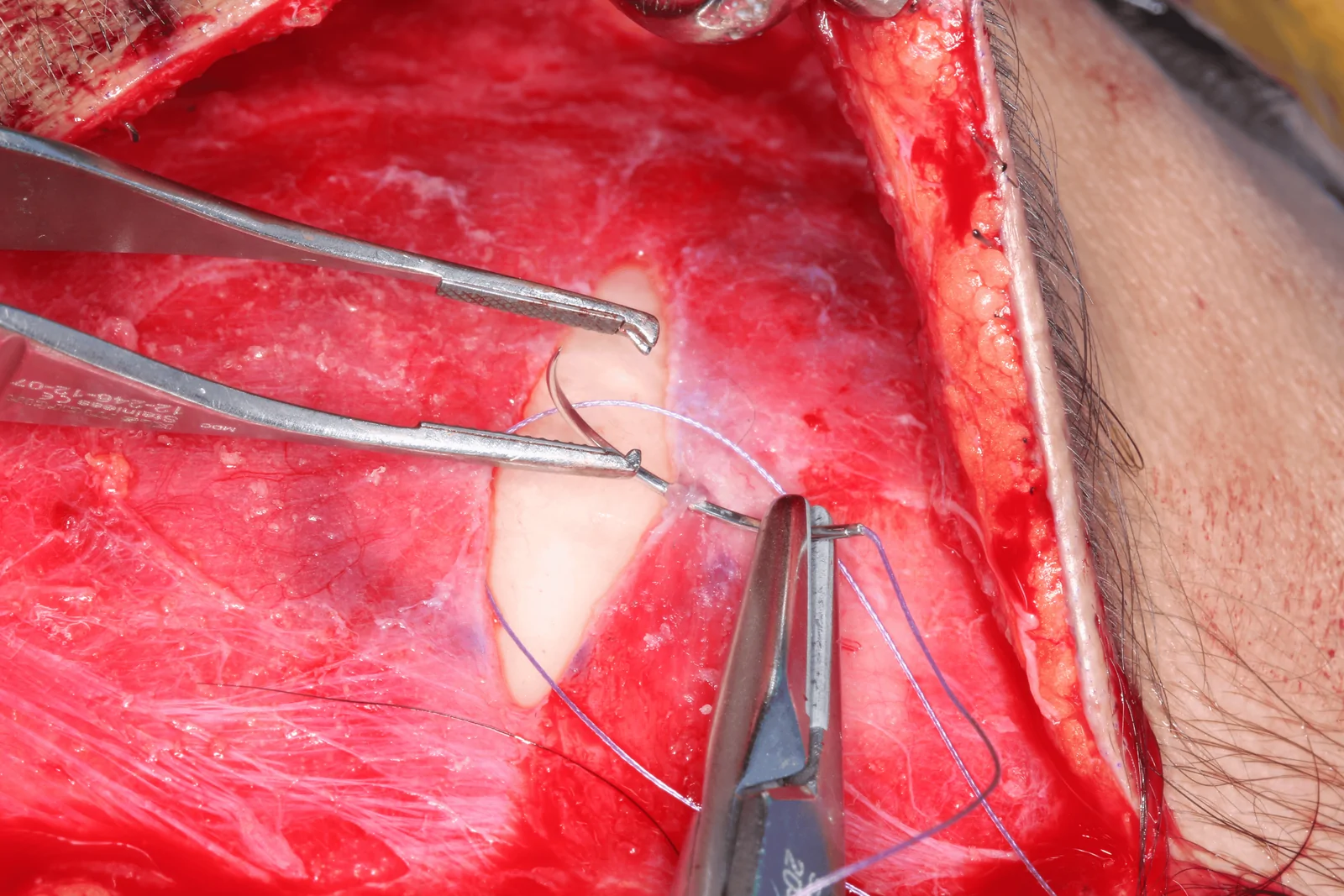
Anatomical Re-approximation of the Frontalis Muscle Following completion of frontal bone contouring, the frontalis muscle was anatomically re-approximated using sutures to restore muscle continuity and re-establish soft-tissue coverage over the contoured frontal bone.

Skin Excision and Closure

With the flap advanced to the desired position, the excess forehead skin proximal to the new hairline was excised, and the wound was closed in layers. Because the flap maintained a consistent thickness throughout its extent, it is expected to redrape more uniformly across the recipient bed during advancement. The absence of abrupt changes in tissue thickness may allow more even distribution of closure forces and could help minimize localized bulging, depressions, or palpable transitions that may otherwise become visible after healing. We propose that this may result in a smoother postoperative forehead contour with less risk of surface irregularity. Figures 6-9 are the intraoperative photographs.

**Figure 6:**
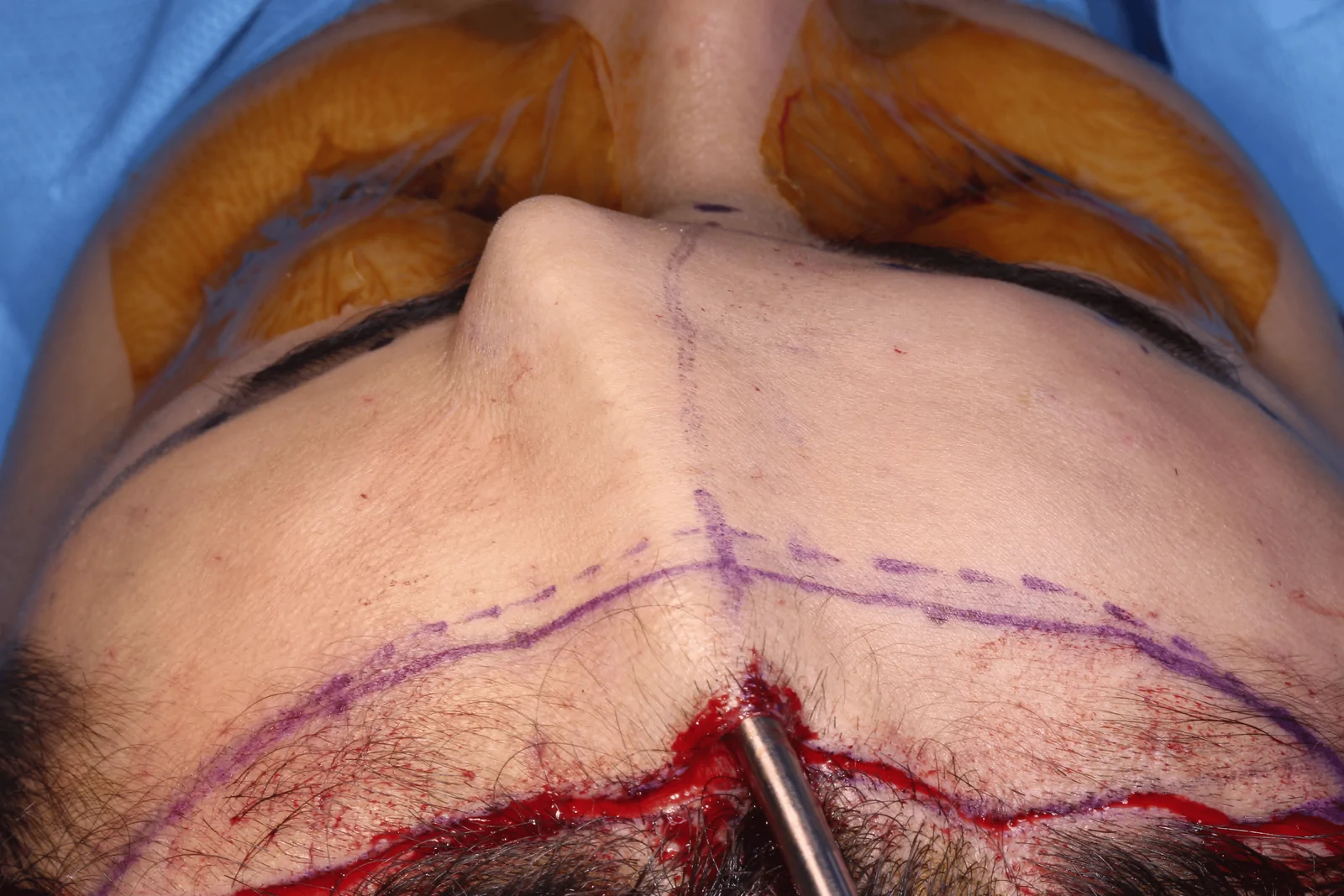
Preoperative Surgical Markings Demonstrating Planned Hairline Advancement Preoperative markings, showing the existing hairline, the planned new hairline, and midline and paramedian vertical reference lines used to guide symmetric advancement.

**Figure 7:**
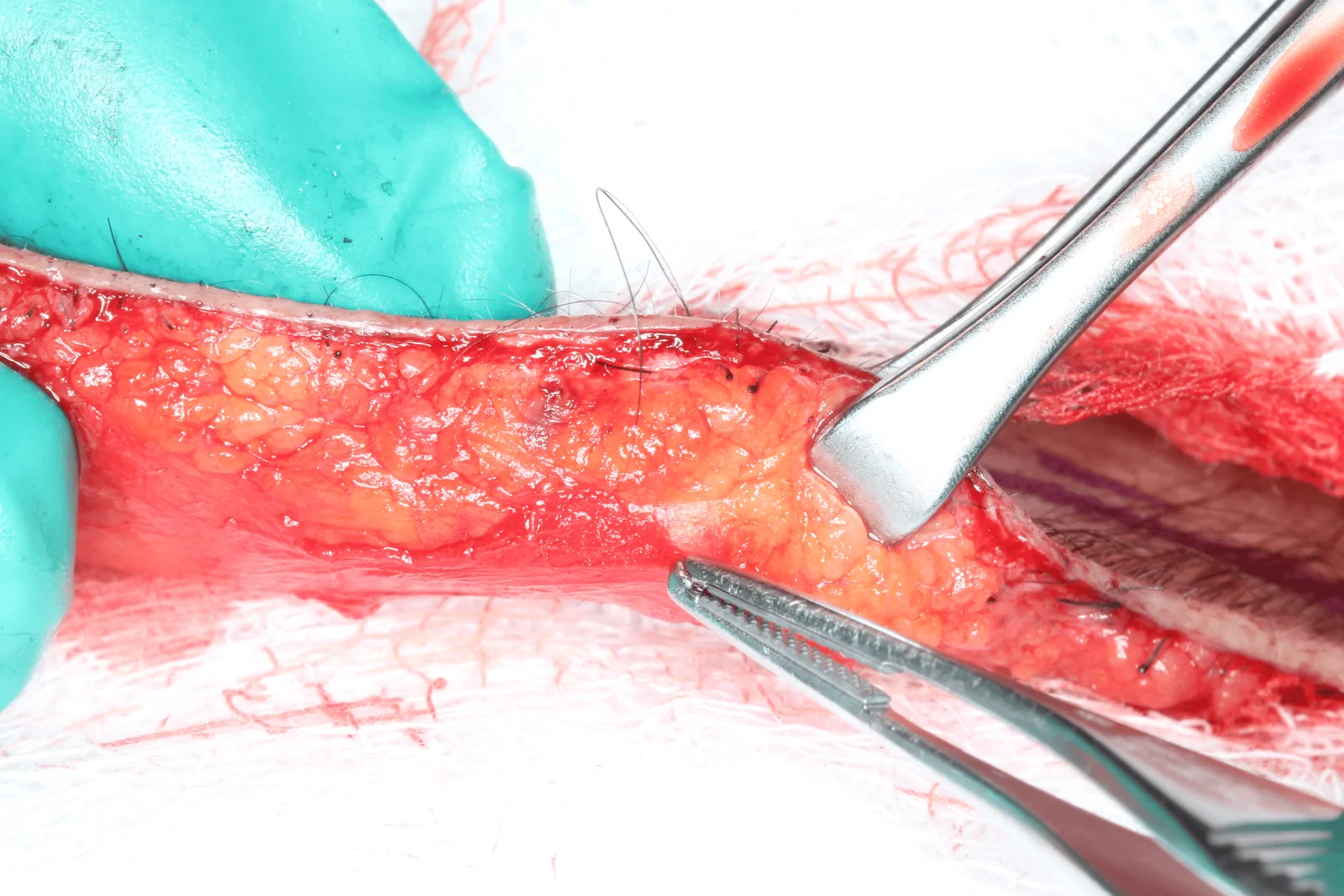
Elevated Flap Edge Following Incision Flap edge at the incision following elevation, demonstrating the skin, subcutaneous tissue, and superficial galeal fascia elevated as a composite flap to expose the desired plane superficial to the frontalis muscle.

**Figure 8:**
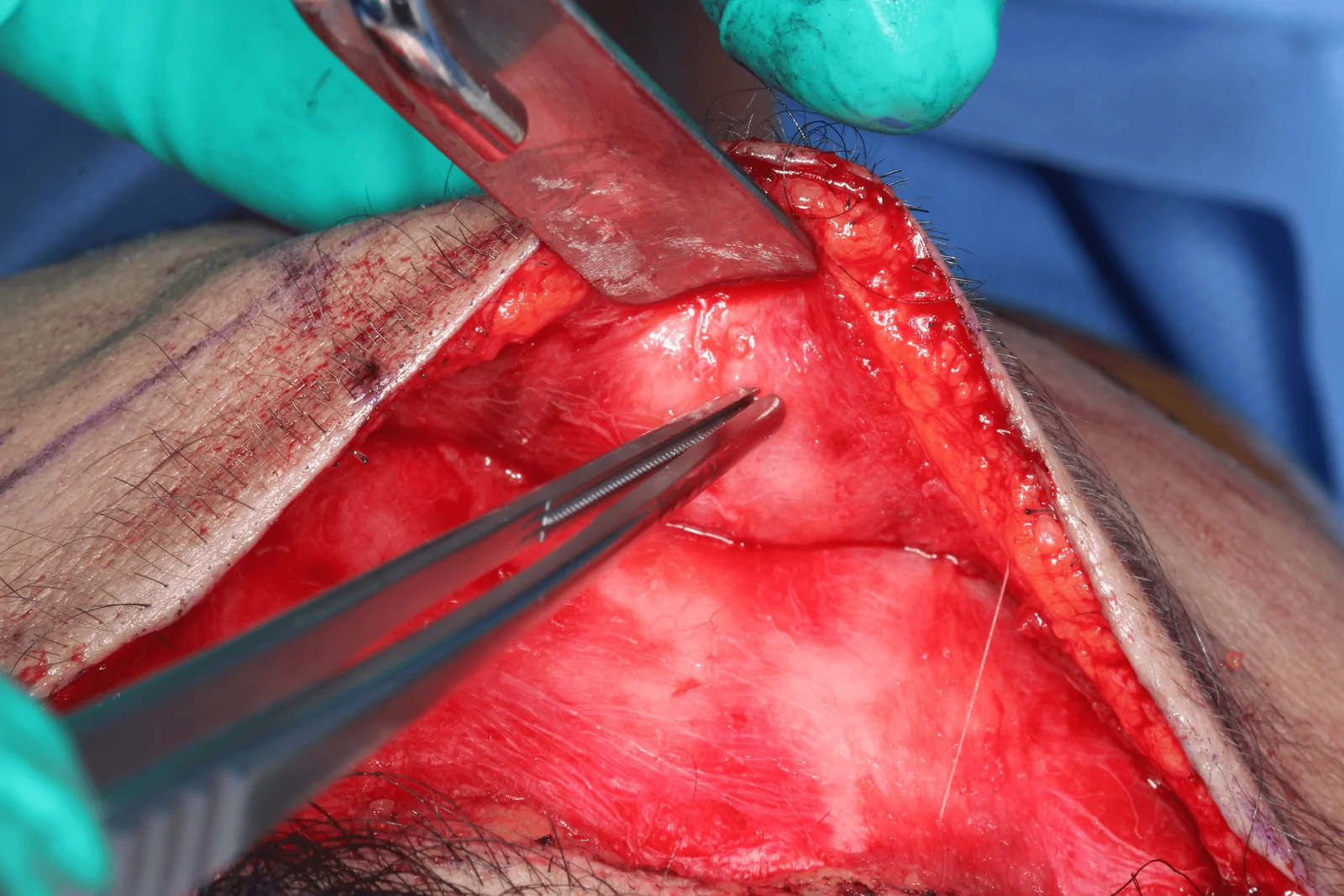
Dissection Plane Superficial to the Frontalis Muscle Dissection maintained in the plane superficial to the frontalis muscle, with the glistening, striated muscle surface visible on the recipient bed as the flap is retracted.

**Figure 9:**
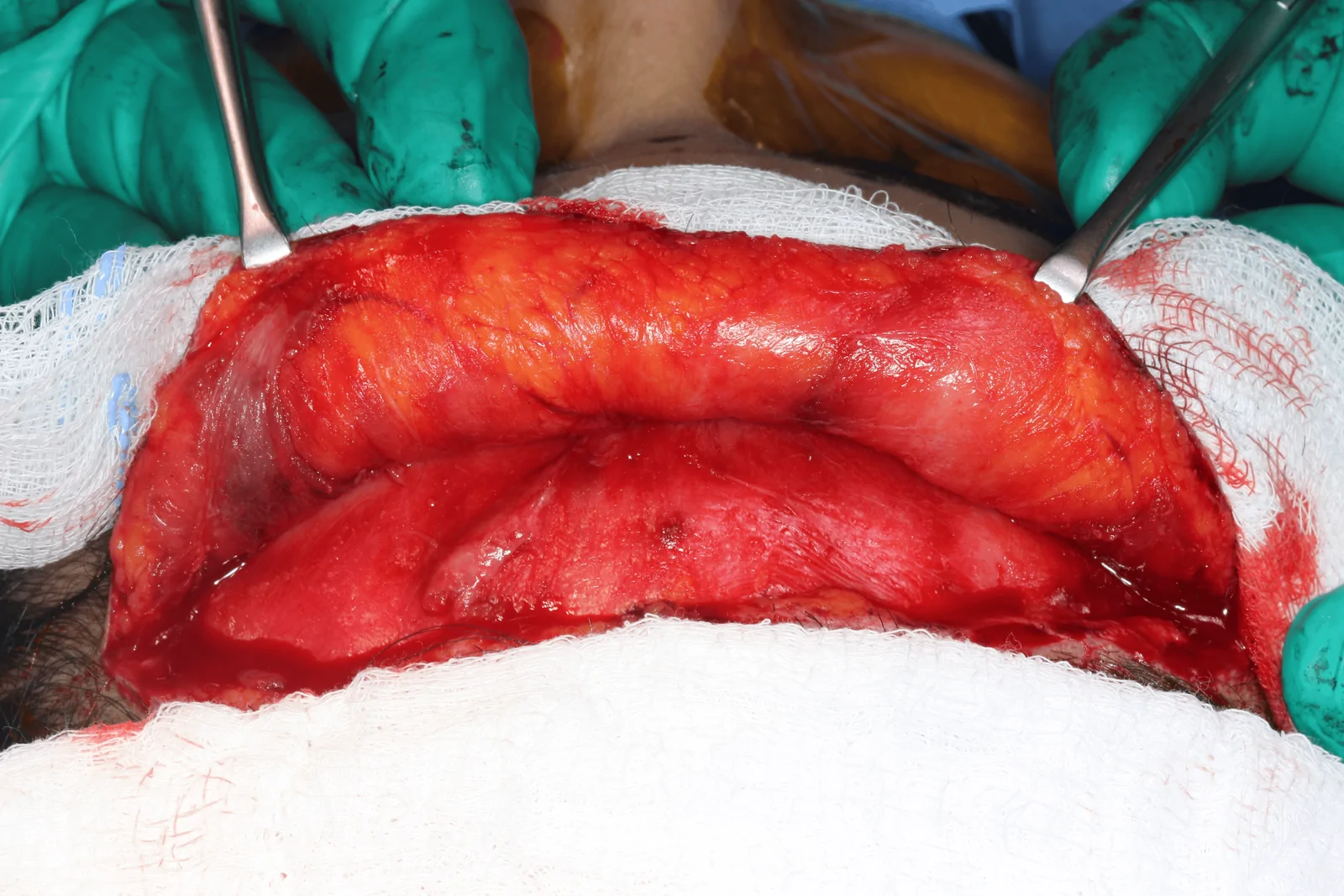
Frontalis Muscle Bed Following Flap Elevation Wide exposure of the frontalis muscle bed following flap elevation, showing the paired muscle bellies on either side of the midline.

## Discussion

This paper describes a refinement to forehead reduction surgery: reliably maintaining the dissection plane superficial to the frontalis muscle across the entire forehead, including through the central bifurcation, where the muscle can't be used as a direct landmark [1]. This specific vulnerability and its link to postoperative contour irregularity have not previously been described in the literature.

The rationale rests on established anatomy: the frontalis muscle varies across the forehead and frequently bifurcates centrally [4], so its surface (the usual landmark for dissection depth) becomes unreliable in these zones, creating a risk of unintentionally variable dissection depth. Our method addresses this by progressing from well-landmarked lateral regions and substituting tactile assessment for direct visualization where the landmark is absent.

The choice of the subsuperficial galea plane itself, rather than a more superficial subcutaneous plane or a deeper submuscular plane, is central to achieving this consistency. Unlike the subcutaneous plane, which lacks a clear, visible tissue landmark and depends on estimated depth alone, the frontalis muscle's glistening, striated surface provides a continuous visual and tactile reference that lets the surgeon confirm dissection depth directly, in real time, across most of the forehead. Drifting more superficially, into the subcutaneous plane, would risk uneven flap thinning, since subcutaneous fat thickness itself varies across the forehead and offers no comparable anatomical checkpoint. Drifting deeper, into a submuscular plane, would place the flap on the far side of the frontalis muscle, adding unnecessary proximity to the periosteum and neurovascular structures and increasing bleeding without any corresponding benefit to flap thickness. Working immediately superficial to the muscle therefore gives the surgeon a reliable checkpoint across most of the forehead, with tactile assessment needed only through the narrow bifurcation zone rather than throughout the entire dissection.

Beyond preserving the muscle itself, we believe the main benefit is a uniformly thick flap, which may redrape more predictably and could reduce the localized tissue-volume differences that can appear as contour irregularities, consistent with general reconstructive surgery principles. While contour irregularity after forehead reduction has not been systematically studied, revision series in facial feminization surgery cite asymmetry and uneven results as reasons for revision [5,6], and we propose inconsistent dissection depth as a plausible, underappreciated contributor. Maintaining a single tissue plane despite a variable landmark is a broader challenge in reconstructive surgery generally; its specific application to the frontalis bifurcation zone is this paper's contribution.

This report has important limitations. As a technical description rather than a clinical outcomes study, we have not collected or reported a formal case series, patient demographics, follow-up duration, complication rates, or objective or patient-reported outcome measures. We have not directly measured flap thickness intraoperatively or postoperatively, nor have we compared outcomes against the conventional subcutaneous dissection technique. The anatomical rationale presented is well established, and the technique is technically reproducible; however, the proposed clinical benefits - a more uniform flap, more predictable redraping, and reduced postoperative contour irregularity - remain hypotheses based on our surgical experience and anatomical reasoning rather than findings demonstrated by this report. A prospective, comparative study with standardised photographic assessment, objective flap thickness measurement, and patient-reported outcomes is needed to test these hypotheses.

## Conclusions

We describe a technique for reliably maintaining the dissection plane superficial to the frontalis muscle throughout forehead reduction surgery, with the aim of reducing the risk of contour irregularities and damage to vital structures. By avoiding inadvertent, inconsistent inclusion of muscle within the flap, the principal advantage of this technique is the creation of a uniformly thick scalp flap through deliberate maintenance of a consistent subsuperficial galea dissection plane. We propose that this homogeneous flap architecture may promote smoother redraping over the recipient bed, may distribute closure forces, and could reduce the likelihood of postoperative contour irregularities while preserving the established principles of conventional forehead reduction surgery. Whether these features translate into improved clinical outcomes requires further investigation.
